# Nerve Growth Factor, Brain-Derived Neurotrophic Factor and Osteocalcin Gene Relationship in Energy Regulation, Bone Homeostasis and Reproductive Organs Analyzed by mRNA Quantitative Evaluation and Linear Correlation Analysis

**DOI:** 10.3389/fphys.2016.00456

**Published:** 2016-10-13

**Authors:** Claudia Camerino, Elena Conte, Maria Cannone, Roberta Caloiero, Adriano Fonzino, Domenico Tricarico

**Affiliations:** ^1^Department of Basic Medical Sciences, Neurosciences and Sense Organs, University of BariBari, Italy; ^2^Department of Molecular and Cellular Physiology, University of CincinnatiCincinnati, OH, USA; ^3^Department of Pharmacy – Drug Sciences, University of BariBari, Italy

**Keywords:** osteocalcin, nerve growth factor, brain-derived neurotrophic factor, gene expression, energy regulation

## Abstract

Nerve Growth Factor (NGF)/Brain-derived Neurotrophic Factor (BDNF) and osteocalcin share common effects regulating energy, bone mass, reproduction and neuronal functions. To investigate on the gene-relationship between NGF, BDNF, and Osteocalcin we compared by RT-PCR the transcript levels of *Ngf, Bdnf* and *Osteocalcin* as well as of their receptors *p75NTR/NTRK1, NTRK2*, and *Gprc6a* in brain, bone, white/brown adipose tissue (WAT/BAT) and reproductive organs of 3 months old female and male mice. Brain and bone were used as positive controls for NGF/BDNF and Osteocalcin respectively. The role of oxitocin(*Oxt*) and its receptor(*Oxtr*) was also investigated. *Ngf* expression shows an opposite trend compared to *Bdnf*. *Ngf* /*p75NTR* expression is 50% higher in BAT than brain, in both genders, but lower in bone. In contrast, *Bdnf* expression in bone is higher than in brain, but low in BAT/WAT. We found *Osteocalcin* gene expressed in brain in both genders, but *Gprc6a* expression is low in brain and BAT/WAT. As expected, *Gprc6a* gene is expressed in bone. *Oxt* gene was markedly expressed in brain, *Oxtr* in the ovaries and in fat and bone in both genders. *Ngf* is highly expressed in reproductive tissues and *p75NTR* mRNA levels are respectively 300, 100, and 50% higher in testis/ovaries/uterus than in brain. In contrast, BDNF genes are not expressed in reproductive tissues. As expected, *Gprc6a* is expressed in testis but not in the ovaries/uterus. A significant correlation was found between the expression levels of the gene ligands and their receptors in brain, BAT and testis suggesting a common pathway of different genes in these tissues in either male and female. Changes in the expression levels of *osteocalcin, Ngf*, or *Bdnf* genes may mutually affect the expression levels of the others. Moreover, it may be possible that different ligands may operate through different receptor subtypes. *Oxt* and *Oxtr* failed to show significant correlation. The up-regulation of *Ngf* /*p75NTR* in BAT is consistent with NGF as an energy regulator and with BDNF regulating bone.

## Introduction

Emerging evidences show that some hormone such as osteocalcin (OST) and the neurotrophins Brain-derived Neurotrophic Factor (BDNF) and Nerve Growth Factor (NGF) as well as oxytocin (Oxt) share common actions suggesting a coordinated regulation (Yano and Chao, 2000; Karsenty, 2011, 2012; Oury et al., 2011, 2013; Camerino et al., 2012; Karsenty and Oury, 2014; Brennan-Speranza and Conigrave, 2015).

The actions of osteocalcin, BDNF and NFG and Oxt are related to the regulation of reproduction, energy and bone homeostasis (Camerino, 2009a,b; Karsenty, 2011; Camerino et al., 2012). Specifically, osteocalcin exists as fully carboxylated or partially carboxylated forms and completely uncarboxylated. The carboxylated osteocalcin has an exclusive effect on bone matrix remodeling stimulating osteoblast formation (Ferron et al., 2010; Karsenty, 2011). The uncarboxylated osteocalcin acts as an hormone and promotes insulin secretion and sensitivity (Fulzele et al., 2010; Karsenty, 2011; Oury et al., 2011). In fact, mice lacking osteocalcin (Ocn)^−/−^ display decreased beta-cell proliferation, glucose intolerance, insulin resistance and increased fat pad (Lee et al., 2007). Uncarboxylated osteocalcin acts on the Gprc6a receptor that activates the AMPc/CREB pathway, which induces the expression of several proteins including testosterone. The *Gprc6a* gene is expressed in skeletal muscle, heart, lung, spleen, kidney, liver, fat, testis and pancreatic beta-cells but it seems absent in the brain. *Gprc6a* is not expressed in the ovary, indicating that the action of osteocalcin on reproductive maturation is gender dependent. The uncarboxylated form of osteocalcin crosses the blood brain barrier (BBB) binds to neurons of the brainstem, midbrain, and hippocampus, enhances the synthesis of monoamine neurotransmitters, inhibits GABA synthesis, prevents anxiety and depression, and favors learning and memory independently of its metabolic functions in mice (Ferron et al., 2010; Oury et al., 2013).

Some of the actions of osteocalcin cannot be easily explained on the basis of the current data. For instance, the neuronal actions of osteocalcin were observed in the absence of expression of the *Gprc6a* gene suggesting that some alternative pathways may play a role in mediating the osteocalcin action in neurons (Oury et al., 2013).

Furthermore, in (Ocn)^−/−^ mice lacking osteocalcin gene, a reduced level of testosterone production was reported in Leydig cells while the circulating levels of LH, the major regulator of testosterone production, were increased 2.5-fold (Yadav et al., 2009; Karsenty, 2011; Oury et al., 2011; Ratto et al., 2012). These data suggest that a compensatory unknown mechanism is functioning in these mice.

The neurotrophins NGF and BDNF besides their well-known classical role in neurogenesis and in synaptic plasticity (Yano and Chao, 2000), are implicated in energy, reproduction and bone metabolism in mice (Rios et al., 2001; Yamashiro et al., 2001; Yao et al., 2002; Camerino et al., 2012). NGF has been reported to play a pivotal role in reproduction inducing for instance Leydig cell maturation (Müller et al., 2006; Ratto et al., 2012). NGF is a potent stimulator of LH secretion, has a dose dependent effect on ovulation and acts via a systemic pathway at physiologically relevant doses. NGF is the ovulation inducing factor (OIF) in seminal plasma; by eliciting LH release, OIF triggers trkA up-regulation and neurite development confirming the NGF-like properties of OIF (Ratto et al., 2012). This peculiar action of NGF in regulating the LH levels may be helpful in those conditions associated with lack of regulatory LH release mechanism as in the case of (Ocn)^−/−^ mice.

Therefore, it may be possible that NGF and BDNF molecules and osteocalcin share common pathways in these tissues, leading to cross talk between different ligand-receptor pathways.

To investigate on the potential relationships between ligands and their receptors, we analyzed by RT-PCR, in the same plate of reaction, the mRNA levels of *Ngf* and associated receptors *Ngfr* (nerve growth factor receptor) and *Ntrk1* (neurotrophic tyrosine kinase, receptor, type 1) genes, of *Bdnf* and associated receptor *Ntrk2* (neurotrophic tyrosine kinase, receptor, type 2) genes, of *Bglap* (osteocalcin), osteocalcin receptor *Gprc6a*, oxytocin *Oxt* and *Oxtr* genes in brain, bone, fat and reproductive organs of 3 months old mice of both genders. In particular, the gene-relationship hypothesis was tested and validated using linear correlation analysis. This statistical approach leads us to compare the gene expression profiles of different tissues in male and female mice. Other methodologies based on protein content evaluation such as ELISA or Western blot do not allow simultaneous multiple comparisons between proteins derived from different tissues in the same plate of reaction.

## Materials and methods

### Animals

Seven male and five female 3 months-old cycle synchronized B6D2 mice were used. The synchronization was performed by separating male and female mice in different cages (three mice per cage) and different rooms and by avoiding contact for at least 96 h before experiments (Pallares and Gonzalez-Bulnes, 2009). The animal care was performed in accordance with the DIRECTIVE 2010/63/EU. The protocol was approved by the Ethics Committee of the University of Bari, 2012, Italy. Animals were deeply anesthetized with an intraperitoneal injection of urethane (1.2 g/kg body weight). The blood was collected from the heart. The animals under anesthesia were sacrified by cervical dislocation. Subsequently, from each animal the following organs where quickly isolated: whole brain, abdominal white adipose tissue (WAT), interscapolar brown adipose tissue (BAT), sexual organs (uterus, ovaries or testicles) and femora. All organs were frozen in liquid nitrogen and stored at −80°C.

### Femora preparation

Femora were dissected from 3-month-old mice and further cleaned from the adhered tissues 3 times in two different ethanol solution both at 70% and then stored in sterile PBS at 4°C. The next day the femora metaphysis were removed and the marrow tissue was eliminated by flushing it with a syringe containing sterile PBS as previously described (Lecka-Czernik, 2012). Cleaned femora were stored at −80°C.

### Real-time PCR experiment

The RNA extraction protocol was chosen based on the amount and type of tissue as previously described (Tricarico et al., 2008; Mele et al., 2014). In particular, total brain, bones (femur), uterus and WAT tissues were extracted with Trizol (Invitrogen); the testis and BAT were extracted with RNeasy Tissue Mini Kit (Qiagen), while the ovaries were extracted with Tissue RNeasy Micro Kit (Qiagen). Total RNA was quantified using a spectrophotometer (ND-1000 NanoDrop, Thermo Scientific, USA). To perform reverse transcription, 400 ng of total RNA was added to 1 μl dNTP mix 10 mM (Roche N.C. 11277049001, Switzerland) and 1 μl Random Hexamers 50 mM (Life Technologies C.N. n808-0127, USA) and incubated at 65°C for 5 min. Afterward, 4 μl 5X First Standard Buffer (Life Technologies C.N. Y02321), 2 μl 0.1 M DTT (Life Technologies C.N. Y00147) and 1 μl l Recombinant RNasin Ribonuclease Inhibitor 40 U/ml (Promega, C.N. N2511, USA) was added and incubated at 42°C for 2 min. One microliter of Super Script II Reverse Trascriptase 200 U/ml (Life Technologies C.N. 18064-014) were added to each solution and incubated at 25°C for 10 min, at 42°C for 50 min and at 70°C for 15 min. All genes were pre-amplificated, because low expressed, by TaqMan PreAmp Master Mix before the real-time experiments. The setup of pre-amplification consisted of 250 ng of reverse-transcribed, 25 μl of the TaqMan PreAmp Master Mix and 12.5 μl of Pool Assay 0.2 × (containing *Hprt1, 2BM, Gapdh, Eef2, Beta-actin, Ngf, Bdnf, Ntrk1, Ntrk2, Ngfr, Bglap, Oxt, Oxtr, Ggcx*). The solution was incubated at 50°C for 2 min, 95°C for 10 min, and for 40 cycles of 95°C for 15 s and 60°C for 1 min. Real-time PCR was performed in triplicate, using the Applied Biosystems Real-time PCR 7500 Fast system. Each reaction was carried in duplicate on a single plex reaction. The PCR was run for 10 min at 95°C and 45 cycle for 0.1 min at 95°C and 0.1 min at 60°C. Each reaction contained 8 ng of cDNA, 0.5 μL Probe TaqMan Gene Expression Assay, 5 μl of TaqMan Universal PCR Master Mix, and nuclease free water for a final volume of 10 μl. The results were compared with a relative standard curve obtained by 6 points of 1:4 serial dilutions. The mRNA expression of the genes was normalized to the best housekeeping genes β*-actin* selected within β*-actin, Hprt1, 2beta-microglobulin, Gapdh*, and *Eef2*. TaqMan Hydrolysis primer and probe gene expression assays were ordered from Applied Biosystems with the following IDs: hypoxanthine guanine phosphoribosyl transferase (*Hprt1*) IDs: Mm 00446968_m1; beta-2 microglobulin (*2bm*) IDs: Mm 00437762_m1; glyceraldehyde-3-phosphate dehydrogenase (*Gapdh*) IDs: Mm 99999915_g1; eukaryotic translation elongation factor 2 (*Eef2*) IDs: Mm 01171434_g1; beta-actin (β*-actin*) (primer For: 5′-CCAGATCATGTTTGAGACCTTCAA-3, primer Rev: 5′-CATACAGGGACAGCACAGCCT-3, probe: VIC-ACC CCA GCC ATG TAC GTA-MGB); bone gamma-carboxyglutamate protein 3 (*Bglap*) IDs: Mm01741771_g1; g protein-coupled receptor, family C, group 6, member A (*Gprc6a*) IDs: Mm01192897_m1; gamma-glutamyl carboxylase *(Ggcx)* IDs:Mm00517274_m1; nerve growth factor (*Ngf*) IDs: Mm 00443039_m1; nerve growth factor receptor (*Ngfr*) IDs: Mm 01309638_m1; neurotrophic tyrosine kinase, receptor, type 1 (*Ntrk1*) IDs: Mm 01219406_m1; brain derived neurotrophic factor (*Bdnf*) IDs: Mm 04230607_s1; neurotrophic tyrosine kinase, receptor, type 2 (*Ntrk2*) IDs: Mm00435422_m1; oxytocin (*Oxt*) IDs: Mm00726655_s1; oxytocin receptor (*Oxtr*) IDs: Mm01182684_m1.

### Carboxylated and uncarboxylated osteocalcin measurement by ELISA assay

Each tissue was homogenized in PBS (10 mg of tissue in 100 μl of PBS) and was cut with micro scissors in the presence of protease inhibitors. After centrifugation at 1500 RPM for 15 min, the supernatants were collected and used for ELISA assays. The levels of carboxylated and uncarboxylated Ost, in the supernatants were measured using duo set enzyme-linked immune assay (ELISA) kits from MyBioSource (San Diego, USA) with MBS039459 and MBS010256 codes, respectively.

### Statistics

All experimental data were expressed as mean ± standard error of the mean (S.E.M.). Differences within and between groups were evaluated using one way ANOVA analisys. Bonferroni test correction to counteract for multiple comparison and test for the significance of each individual hypothesis at a significant level of α = 0.05 with GraphPad Prism version 6 software was also performed. Linear correlation analysis was performed between gene expression data to calculate the coefficient of correlation (*r*) by solving the equation *y* = *b*_0_ + *b*_1_*x*_1_ using Excel software (Microsoft, U.S.A.) as previously described (Tricarico et al., 2010).

## Results

The transcript levels of all the four factors and their relative receptors were measured in different tissues of male and female mice using a unique plate of reaction.

**Figure 1:**
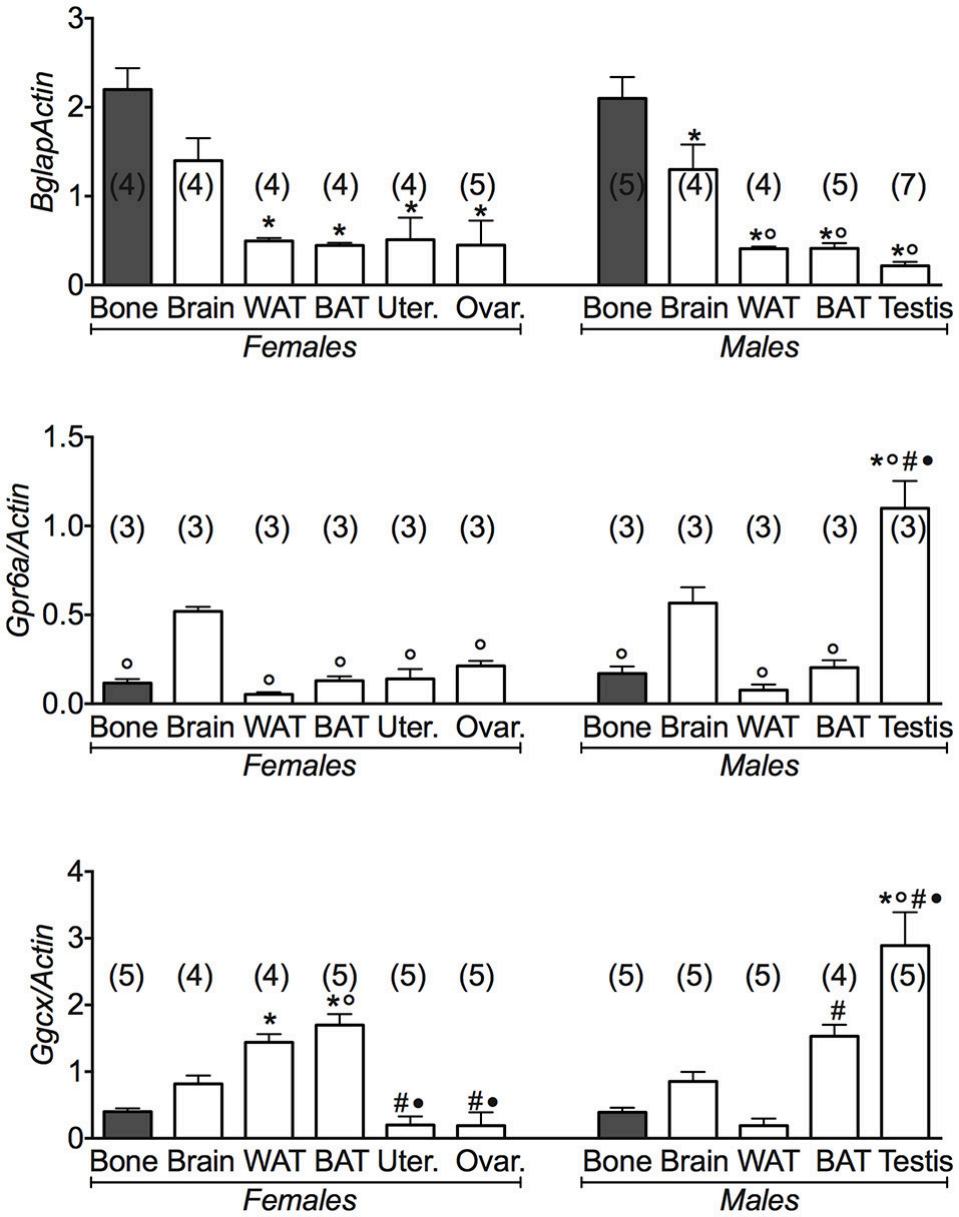
**Osteocalcin genes and their receptors in different tissues of 3 months old female and male mice**. Relative expression levels of the osteocalcin genes in the tissues of 3 months old female and male mice. The genes under investigations were: the osteocalcin *Bglap* gene and its receptor *Gprc6a* and the gamma-glutamyl carboxylase *Ggcx* gene. The tissues under investigations were: brain, bone, white adipose tissue (WAT), brown adipose tissue (BAT), uterus, ovaries, and testis. Brain and bone data were reported for comparison. Bone has to be considered the positive control of *Bglap* gene expression. In each graph, the bars are the means ± SEM from the *n* animals indicated in brackets. For the *Gprc6a* gene, 2 out of 5 and 4 out of 7 analyzed samples from female and male mice, respectively, were not amplified due to the extremely low expression levels of this gene in all tissues except testis. Differences were found within groups and between groups as determined by one way analysis of variance for *Bglap, Gprc6a*, and *Ggcx*. Data significantly different (*P* < 0.05) vs. brain (^◦^), bone (^*^), WAT (^#^), and BAT (^•^) using Bonferroni analysis.

Using bone data as positive control for osteocalcin gene and its receptor *Gprc6a*, we found that osteocalcin gene was markedly expressed also in brain and less in WAT and BAT and reproductive organs, in both male and female mice (Figure 1). A large variance ratio was calculated indicating differences within groups and between groups as determined by one way analysis of variance for *Bglap* (*F* = 11.04 female; *F* = 29.22 male), *Gprc6a* (*F* = 5.1 female; *F* = 13.49 male) and *Ggcx* (*F* = 21.1 female; *F* = 18.49 male). The rank order of tissue expression of the *osteocalcin* gene in both male and female mice was bone ≥ brain > WAT = BAT ≥ reproductive organs. In agreement with previous reports (Oury et al., 2011, 2013), in our experiments the osteocalcin receptor *Gprc6a* was expressed in testis. An extremely low expression of the receptor gene was observed in the ovaries, uterus, and fat tissues in agreement with other reports (Karsenty, 2011, 2012; Karsenty and Oury, 2014; Figure 1).

**Figure 2:**
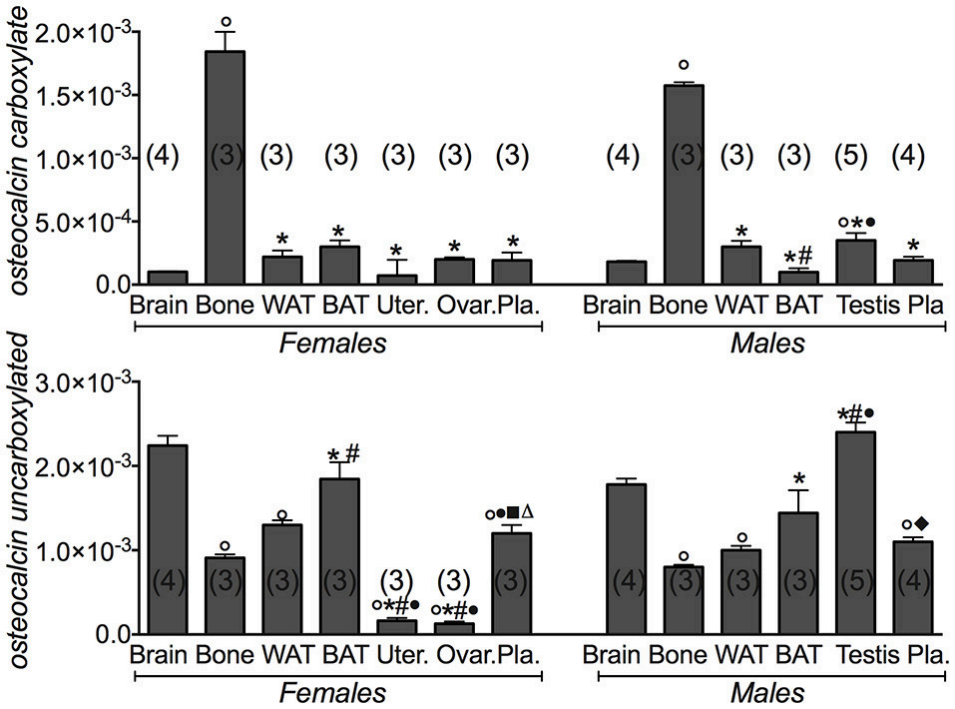
**Carboxylated osteocalcin and uncarboxylated osteocalcin proteins in the tissues of 3 months old female and male mice**. Protein levels of the carboxylated osteocalcin and uncarboxylated osteocalcin in the tissues of 3 months old female and male mice. The tissues under investigations were: brain, bone, white adipose tissue (WAT) and brown adipose tissue (BAT), uterus, ovaries, testis and plasma. In each graph, the bars are the means ± SEM from the *n* animals indicated in brackets. Differences were found within groups and between groups as determined by one way analysis of variance for carboxylated osteocalcin and uncarboxylated osteocalcin. Data significantly different (*P* < 0.05) vs. brain (^◦^), bone (^*^), WAT (^#^), BAT (^•^), (■) ovaries, and (♦) testis using Bonferroni analysis.

We additionally investigated the expression levels of the gamma-glutamyl carboxylase (*Ggcx*) gene involved in the decarboxylation of several proteins including osteocalcin. We found a significant expression of this gene in fat tissues, brain and testis. A large variance ratio was calculated indicating differences within groups and between groups as determined by one way analysis of variance for carboxylated osteocalcin (*F* = 66 female; *F* = 24.67 male) and uncarboxylated osteocalcin (*F* = 62.16 female; *F* = 251 male). The rank order of tissue expression of *Ggcx* gene in male mice was: testis > BAT > brain > bone = WAT; in female was BAT ≥ WAT > brain > bone > reproductive organs (Figure 1). Furthermore, ELISA assay showed a significant level of the carboxylated osteocalcin in bone of both male and female mice, according to the role of this type of protein in bone homeostasis, while the uncarboxylated form predominates in all other tissues (Figure 2). The uncarboxylated protein crosses the BBB and acts on its receptor, according to the role of this protein as a peripheral signal released from bone and acting on other tissues (Oury et al., 2013). Despite the high expression of *osteocalcin* gene in brain (Figure 1), the level of the carboxylated osteocalcin is low, as demonstrated by ELISA experiments (Figure 2). According to previous reports (Karsenty, 2011; Oury et al., 2011; Karsenty and Oury, 2014), a low level of this protein was found in the uterus and ovaries (Figure 2).

**Figure 3:**
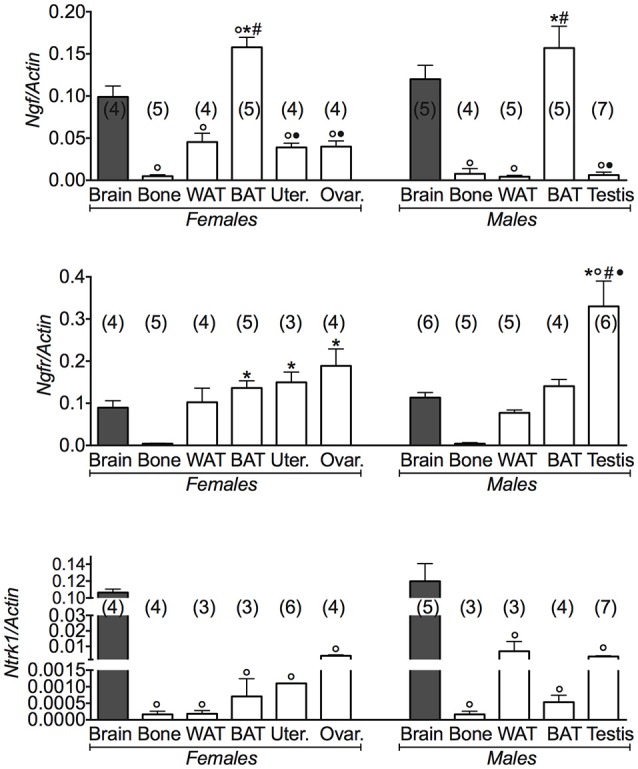
**NGF gene and its receptors in different tissues of 3 months old female and male mice**. Relative expression levels of NGF gene and its receptors in brain, bone, white adipose tissue (WAT) and brown adipose tissue (BAT), uterus, ovaries, testis of 3 months old female and male mice. The genes under investigations were: nerve growth factor *Ngf* and associated receptor nerve growth factor receptor *Ngfr*, and neurotrophic tyrosine kinase, receptor, type 1 *Ntrk1*. Brain data were reported for comparison as a positive control. In each graph, the bars are the means ± SEM from the *n* animals indicated in brackets. For the *Ntrk1* gene, 2 out of 5 analyzed samples in WAT and BAT from female mice, and 4 out of 7 analyzed samples in bone and WAT from male mice were not amplified due to the extremely low expression levels of this gene in these tissues. Differences were found within groups and between groups as determined by one way analysis of variance for *Ngf*, *Ngfr*, and *Ntrk1*. Data significantly different (*P* < 0.05) vs. brain (^◦^), bone (^*^), WAT (^#^), and BAT (^•^) using Bonferroni analysis.

We found that the mRNA levels of *Ngf* and *Ngfr* genes were markedly elevated in BAT other than in brain in either female and male mice (Figure 3). The brain data were used as positive controls for *Ngf* and *Bdnf* gene expression. A large variance ratio was calculated indicating differences within groups and between groups as determined by one way analysis of variance for *Ngf* (*F* = 41.31 female; *F* = 29.24 male), *Ngfr* (*F* = 7.6 female; *F* = 15.02 male), and *Ntrk1* (*F* = 630.5 female; *F* = 23.18 male). The rank order of expression of *Ngf* genes was comparable in either gender and was: BAT > brain > WAT > reproductive tissues > bone. The non-specific receptor gene *Ntrk1* was more expressed in brain than in BAT and WAT showing a low expression in bone in both genders (Figure 3). These findings are consistent with our hypothesis of NGF as a regulator of energy, playing a minor role in bone.

The *Ngfr* (p75NTR) receptor gene was significantly expressed in the reproductive tissues in female and male mice, in this context the expression of *Ngfr* (p75NTR) receptor gene in the testis was 300% higher than in brain (Figure 3).

**Figure 4:**
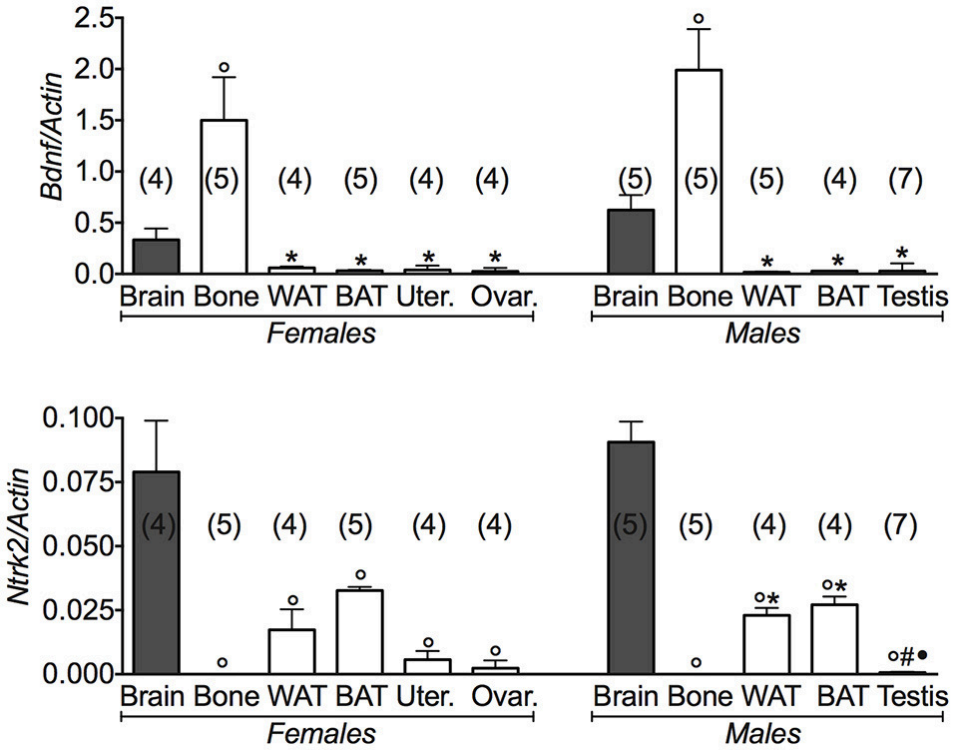
**BDNF gene and its receptor in different tissues of 3 months old female and male mice**. Relative expression levels of brain derived neurotrophic factor (*Bdnf*) and its receptor in brain, bone, white adipose tissue (WAT) and brown adipose tissue (BAT), uterus, ovaries, testis of 3 months old female and male mice. The genes under investigations were: brain derived neurotrophic factor *Bdnf* and associated neurotrophic tyrosine kinase, receptor, type 2*Ntr2*. Brain data were reported for comparison as positive control. In each graph, the bars are the means ± SEM from the *n* animals indicated in brackets. Differences were found within groups and between groups as determined by one way analysis of variance for *Bdnf* and *Ntrk2*. Data significantly different (*P* < 0.05) vs. brain (^◦^), bone (^*^), WAT (^#^), and BAT (^•^) using Bonferroni analysis.

Conversely, the expression levels of the *Bdnf* gene were significantly higher in bone than brain (Figure 4). A large variance ratio was calculated indicating differences within groups and between groups as determined by one way analysis of variance for *Bdnf* (*F* = 8.9 female; *F* = 20.4 male), and *Ntrk2* (*F* = 12.74 female; *F* = 95.44 male). The rank order of expression of the *Bdnf* gene in either gender was indeed: bone > brain >> WAT and BAT. The expression profile of the receptor gene was inversely related to that of the *Bdnf* gene in the same tissues, a result that is expected for a very specific ligand/receptor pathway (Figure 4). The rank order of expression of *Ntrk2* gene in either gender was indeed brain > BAT = WAT >> bone. These findings showed that the BDNF pathway plays a major role in bone, playing a minor role in the reproductive tissues.

Thus, it appears that the neurotrophins have distinct peripheral roles: the NGF pathway seems to regulate mostly energy balance and reproduction, while BDNF pathway is important for bone homeostasis.

**Figure A1:**
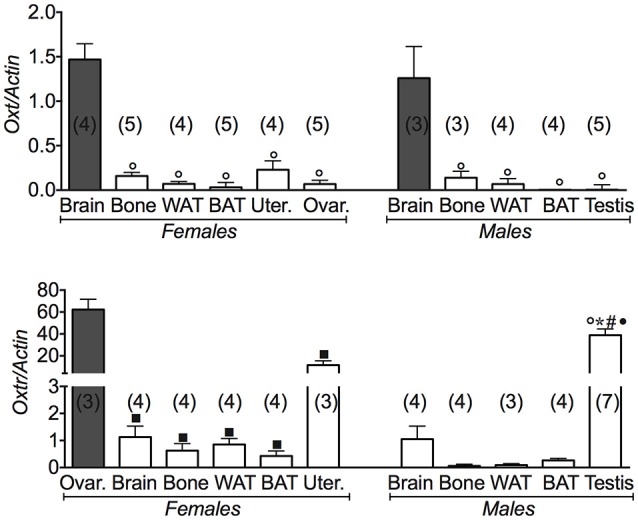
**Oxytocin gene and its receptor in different tissues of 3 months old female and male mice**. Relative expression levels of the oxytocin gene and its receptor in brain, bone, white adipose tissue (WAT) and brown adipose tissue (BAT), uterus, ovaries and testis of 3 months old female and male mice. Ovaries data were used as positive control for *Oxt* and *Oxtr*. The genes under investigations were: the oxytocin (*Oxt)* and oxytocin receptor (*Oxtr)* gene. In each graph, the bars are the means ± SEM from the *n* animals indicated in brackets. Differences were found within groups and between groups as determined by one way analysis of variance for *Oxt*, and *Oxtr*. Data significantly different (*P* < 0.05) vs. brain (^◦^), bone (^*^), WAT (^#^), BAT (^•^), and (■) ovaries using Bonferroni analysis.

We next investigated the expression levels of Oxt and its receptor in the same tissues (Figure A1). We found that the *Oxt* gene was markedly expressed in brain and less expressed in bone; *Oxtr* was also expressed in fat tissues and bone in both genders, although at a lower level. In the reproductive tissues, *Oxtr* was markedly expressed in ovaries. The ovaries data were considered as a positive control of *Oxt* and *Oxtr* gene expression. A large variance ratio was calculated indicating differences within groups and between groups as determined by one way analysis of variance for *Oxt* (*F* = 42.2 female; *F* = 14.62 male), and *Oxtr* (*F* = 48.67 female; *F* = 22.6 male).

The potential relationship between osteocalcin *Bglap, Ngf, Bdnf*, and *Oxt* genes and their respective gene receptors *Gprc6a, Ngfr/Ntrk1, Ntrk2, Oxtr* was investigated by a linear correlation analysis focusing on gene expression data collected from different tissues. The gene expression data obtained in each tissue were fitted using a linear regression equation (see Materials and Methods). An high level of correlation was calculated in brain, BAT and in testis for NGF, BDNF, and osteocalcin (Table 1). The *Oxt/Oxtr* gene expression data were responsible for the loss of correlation or low correlation calculated in the tissues other than brain and BAT. These findings suggest a tissues specific correlation between the genes expressing ligands and their receptors in the investigated tissues.

**Table 1:** **Linear correlation analysis of genes expression in different tissues in female and male mice**.

**Genes: *Bglap-Ngf-Ngf-Bdnf*- vs. *Gprc6a-Ngfr-Ntrk1-Ntrk2-***	**Equations**	**Correlation factor *R*^2^**
Brain	Male *y* = 0.5329x − 0.0156	0.917
	Female *y* = 0.6929x − 0.054	0.8908
WAT	Male *y* = 0.5896x + 0.3755	0.5446
	Female *y* = 0.1055x + 0.0376	0.5105
BAT	Male *y* = −0.5757x + 0.249	0.776
	Female *y* = 0.485x + 0.0209	0.8922
Bone	Male *y* = −0.1929x + 0.229	0.4505
	Female *y* = 0.0637x − 0.0028	0.3462
Testis	*y* = −2.185x + 0.557	0.7181
Ovaries	*y* = −29.607x + 20.221	0.4911
Uterus	*y* = 5.3416x + 3.9574	0.4712

*Linear correlation analysis of gene expression was performed between the genes expressing ligands and their respective receptors in different tissues. An high correlation with R^2^ >0.5 was calculated in brain, BAT and testis in either male and female mice. The sample size was between 3 and 7 for each group*.

## Discussion

In the present work we compared the expression profile of the *Ngf/Bdnf/Oxt-Osteocalcin* genes and their receptors in different tissues of 3 months old male and female mice in the same plate of reaction.

We found a significant linear correlation between the expression levels of the hormone genes *Bglap-NGF-BDNF* and their receptors *Gprc6a-Ngfr/Ntrk1-Ntrk2* in brain and BAT in either male and female. It is therefore expected that changes in the expression levels of *osteocalcin, Ngf* and *Bdnf* genes may mutually affect the expression levels of the others in brain and BAT. Taking into account also the effects of osteocalcin in male fertility, osteocalcin may act in a “male specific estrogen-like” manner.

The *Oxt/Oxtr* genes in our experiments were, however responsible for the loss of correlation in different tissues.

**Figure 5:**
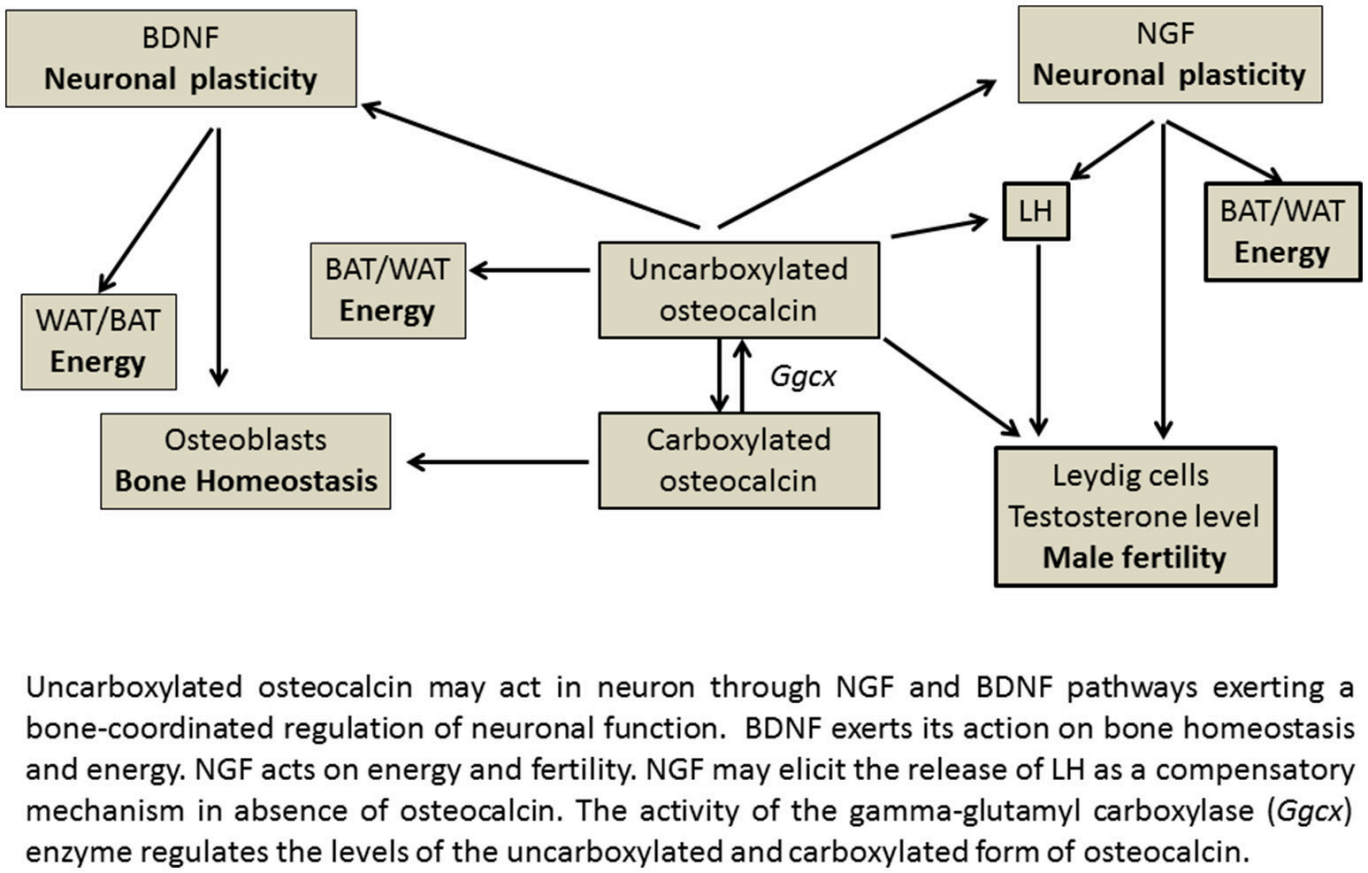
**Gene-relationship between osteocalcin and neurothrophin in energy regulating organs**.

The relationship between neurotrophins *Ngf* or *Bdnf* genes and *osteocalcin* gene found in our experiments may help to explain the actions of osteocalcin in those brain areas lacking the osteocalcin receptor *Gprc6a*. It is likely that neurotrophins can mediate most of the actions of osteocalcin in various brain areas, transducing the peripheral signaling represented by the uncarboxylated osteocalcin that crosses the BBB into neuronal signaling (Figure 5).

In this context, we propose that NGF may have a distinct compensatory role in regulating reproduction and energy balance of BAT and increasing LH levels in the absence of osteocalcin, thereby explaining the enhanced LH levels observed in the (Ocn)^−/−^ mice (Yadav et al., 2009; Karsenty, 2011; Oury et al., 2011; Ratto et al., 2012). Interestingly the *Ngfr* (p75NTR) receptor gene is expressed in testis at levels greater than brain. This matches the expression of *Gprc6a* genes. In our experiments the NGF genes were indeed highly expressed in the reproductive tissues. NGF has been reported to plays a pivotal role in reproduction inducing for instance Leydig cell maturation (Müller et al., 2006; Ratto et al., 2012). NGF is a potent stimulator of LH secretion, has a dose dependent effect on ovulation and acts via a systemic pathway at physiological relevant doses. NGF is the OIF in seminal plasma; by eliciting LH release, OIF triggers TrkA up-regulation and neurite development confirming the NGF-like properties of OIF (Ratto et al., 2012).

These observations imply that these hormones may share common pathways in tissues, leading to cross talk between different ligand-receptor pathways. The existence of alternative pathways for the hormonal actions has been described for estrogen and androgen that act through genomic and non-genomic pathways (Falkenstein et al., 2000; Camerino, 2012; Karsenty and Oury, 2012; Prossnitz and Barton, 2014). The biological advantage of this mechanism is that the lack of effects of a specific gene following pathophysiological conditions or gene downregulation can be counterbalanced by the action of other genes (Karsenty and Oury, 2012).

*Ngf* and *Bdnf* gene expression appears to show an opposite trend, with NGF regulating reproduction and energy in both genders and BDNF regulating bone homeostasis and energy (Figure 5). The *Bdnf* and *Ntrk2* genes are indeed not expressed in the reproductive organs in our experiments, in contrast to *Ngf*, *Ngfr, Ntrk1* genes, and *Oxtr* gene which are expressed in these tissues (Dissen et al., 2009; Melmed et al., 2011; Ratto et al., 2012). The *Bdnf* and *Ntrk2* genes are instead highly expressed in bone. In agreement with this conclusion, previous work demonstrated that brain-targeted BDNF conditional knockout mice (Bdnf(2lox/2lox)/93), in which the *Bdnf* gene has been specifically deleted in brain, show high bone mass and are a metabolic phenocopy of the leptin deficient ob/ob mice, but independent of adrenaline and serotonin pathway (Ducy et al., 2000; Yadav et al., 2009; Camerino et al., 2012).

In regard to the expression of the osteocalcin receptor *Gprc6a* gene, our data are in line with other reports showing elevated levels of the mRNA of this gene in testis and extremely low levels in ovaries (Oury et al., 2011; Karsenty, 2012). However, the investigation of the role of Gprc6a in regulation of bone mass accrual has generated contradicting findings (Pi et al., 2005; Wellendorph et al., 2009; Karsenty, 2012). In line with these findings, our data reported extremely low levels of the related mRNA in bone.

The *Ngfr* (p75NTR) receptor gene was significantly expressed in the reproductive tissues in female and male mice suggesting that the gonads receive NGF signaling released from CNS through NGF receptors *p75NTR/NTRK1*. BDNF signaling may also contributes to this cascade of reaction through p75NTR albeit with a low affinity (Sandhya et al., 2013).

Therefore, NGF may act as a physiologic mediator of osteocalcin both centrally through the sprouting of new synapses, and in energy regulation, and peripherally probably regulating steroid production in Leydig cells in the testosterone deficient Ost^−/−^ mice (Oury et al., 2013).

Should be underlined, that we measured mRNA levels and no functional proteins were studied. The mRNA levels in our work were extracted from whole organs with no evaluation of cell specific expression of the investigated genes. However, here we investigated on the potential correlation between genes by using a multiple comparison of mRNA levels of various tissues collected from female and male mice in the same plate of reaction. In addition, the high sensitivity of the RT-PCR technique in comparison to other methodology allows the determination of very small quantity of mRNA in the tissues in which some genes of interest may show extremely low level of expression while protein may be undetectable in the same sample.

In the present work, we focused our attention on young adult mice to keep sample homogeneous but in the future work it can be interesting to evaluate the gene-relationship hypothesis even during development. Future experiments will further confirm our hypothesis in animal models.

## Author contributions

CC, elaborated the hypothesis and theory; CC and DT designed the studies; EC, MC, and RC conducted the experiments; CC and EC analyzed the results; CC and DT wrote the manuscript and all authors approved the manuscript.

### Conflict of interest statement

The authors declare that the research was conducted in the absence of any commercial or financial relationships that could be construed as a potential conflict of interest.
